# Development and Evaluation of a Self-Nanoemulsifying Drug Delivery System for Sinapic Acid with Improved Antiviral Efficacy against SARS-CoV-2

**DOI:** 10.3390/pharmaceutics15112531

**Published:** 2023-10-25

**Authors:** Hani A. Alhadrami, Ahmed S.G. Srag El-Din, Hossam M. Hassan, Ahmed M. Sayed, Albaraa H. Alhadrami, Mostafa E. Rateb, Demiana M. Naguib

**Affiliations:** 1 Faculty of Applied Medical Sciences, Department of Medical Laboratory Technology, King Abdulaziz University, P.O. Box 80402, Jeddah 21589, Saudi Arabia; hanialhadrami@kau.edu.sa; 2 King Fahd Medical Research Centre, King Abdulaziz University, P.O. Box 80402, Jeddah 21589, Saudi Arabia; 3 Molecular Diagnostics Laboratory, King Abdulaziz University Hospital, P.O. Box 80402, Jeddah 21589, Saudi Arabia; 4 Department of Pharmaceutics, Faculty of Pharmacy, Delta University for Science & Technology, Gamasa City 35712, Egypt; ahmed.serageldin@deltauniv.edu.eg; 5 Department of Pharmacognosy, Faculty of Pharmacy, Nahda University, Beni-Suef 62513, Egypt; hossam.mokhtar@nub.edu.eg (H.M.H.); ahmed.mohamed.sayed@nub.edu.eg (A.M.S.); 6 Department of Pharmacognosy, Faculty of Pharmacy, Beni-Suef University, Beni-Suef 62514, Egypt; 7 School of Computing, Engineering & Physical Sciences, University of the West of Scotland, Paisley PA1 2BE, UK; 8 Department of Pharmaceutics, Faculty of Pharmacy, Nahda University (NUB), Beni-Suef 62513, Egypt

**Keywords:** self-nanoemulsifying drug delivery system, sinapic acid, SARS-CoV-2, *G*aMD, M^pro^

## Abstract

This study aimed to develop a self-nanoemulsifying drug delivery system (SNE) for sinapic acid (SA) to improve its solubility and antiviral activity. Optimal components for the SA-SNE formulation were selected, including Labrafil as the oil, Cremophor EL as the surfactant, and Transcutol as the co-surfactant. The formulation was optimized using surface response design, and the optimized SA-SNE formulation exhibited a small globule size of 83.6 nm, high solubility up to 127.1 ± 3.3, and a 100% transmittance. In vitro release studies demonstrated rapid and high SA release from the formulation. Pharmacokinetic analysis showed improved bioavailability by 2.43 times, and the optimized SA-SNE formulation exhibited potent antiviral activity against SARS-CoV-2. The developed SA-SNE formulation can enhance SA’s therapeutic efficacy by improving its solubility, bioavailability, and antiviral activity. Further in silico, modeling, and Gaussian accelerated molecular dynamics (*G*aMD)-based studies revealed that SA could interact with and inhibit the viral main protease (M^pro^). This research contributes to developing effective drug delivery systems for poorly soluble drugs like SA, opening new possibilities for their application via nebulization in SARS-CoV-2 therapy.

## 1. Introduction

Sinapic acid (SA) is a natural organic compound belonging to the phenolic acid family [1]. It is abundant in various plant sources, including fruits, vegetables, grains, and spices [2]. SA is synthesized by plants from the amino acid phenylalanine through the phenylpropanoid pathway [3]. It is involved in several biological processes in plants, such as UV protection, cell wall lignification, and defense against pathogens and oxidative stress [1,4]. In addition to its role in plants, SA has been studied extensively for its potential health benefits in humans. It exhibits potent antioxidant [5], anti-inflammatory [6], and anticancer properties [7]. It also shows promising effects on various health conditions, including diabetes [8], cardiovascular diseases [9], and neurodegenerative disorders [10]. It was also confirmed earlier during the coronavirus pandemic that SA selectively inhibited SARS-CoV-2 replication by targeting its envelope protein [11]. 

The body readily absorbs SA, and it can be found in the bloodstream after consuming foods containing it [12]. It can also be obtained through dietary supplements [12]. However, more research is needed to determine the optimal dose and long-term safety of SA supplementation. Also, SA has poor solubility in water, which limits its therapeutic efficacy and bioavailability [13]. A self-nanoemulsifying drug delivery system (SNEDDS) has been used to solve these problems.

An SNEDDS is a drug delivery system that uses a mixture of oils, surfactants, and co-surfactants to form a fine oil-in-water nano-emulsion when exposed to aqueous media, such as gastrointestinal fluids. This system is thermodynamically stable and can enhance the solubility and bioavailability of poorly soluble drugs, thereby improving their therapeutic efficacy [14]. An SA self-nano-emulsion (SA-SNE) is a promising drug delivery system that can enhance SA’s solubility, absorption, and bioavailability. The development of an SA-SNE requires the optimal choice of oils, surfactants, and co-surfactants. The choice of these components is critical in achieving a stable and effective self-nanoemulsifying formulation. The oils, surfactants, and co-surfactants selected for this study were chosen based on data collected from the literature [15,16,17]. The selection of oils is pivotal in an SNEDDS as they play a central role in stabilizing the emulsion and determining its properties. The oils chosen for this study were Labrafil, Capryol-90, Labrafac, olive oil, anise oil, almond oil, soybean oil, and corn oil. These oils were selected because they are known to be effective emulsifiers capable of forming oil-in-water (O/W) emulsions upon contact with an aqueous phase [18]. Each of these oils has unique characteristics that can influence the emulsion’s stability, viscosity, and compatibility with various active ingredients [19,20]. 

Surfactants are essential for emulsion stability, reducing interfacial tension between oil and water phases. This study employed hydrophilic non-ionic surfactants, including Cremophor El, Tween 20, and Tween 80. The choice of these surfactants is supported by their high hydrophilic–lipophilic balance (HLB) values, which indicate their ability to stabilize O/W emulsions effectively [21,22,23]. Furthermore, their low oral toxicity is crucial for pharmaceutical and cosmetic applications, where safety is a paramount concern [24,25]. Co-surfactants are often used with surfactants to enhance an emulsion’s stability and modify its properties. The co-surfactants selected for this study were Labrasol, Transcutol, PEG 400, and propylene glycol. These co-surfactants were chosen based on their ability to improve an emulsion’s droplet size distribution, viscosity, and overall stability [26,27]. Their compatibility with the chosen oils and surfactants makes them suitable for creating emulsions with specific desired characteristics.

The current study’s aim was to formulate an SA-SNE with a careful selection of its components, oils, surfactants, and co-surfactants. The optimization process was performed using Design Expert software (Version 11.1.2.0). The safety and efficacy of the SA-SNE in clinical situations were evaluated via pharmacokinetic study in rabbits through nebulization of the prepared SA-SNE mist and in vitro antiviral activity study against SARS-CoV-2. SA’s mode of action was investigated via in vitro-based and in silico-based experiments, including MD simulations. In this context, we present a novel SNE system that produces nano-emulsion mists for pulmonary SA delivery via nebulization. 

## 2. Materials and Methods

### 2.1. Screening of Components for SA-SNE Development

The equilibrium solubility of SA was studied in various oils, surfactants, and cosurfactants. Oils of different saturation degrees (medium- or long-chain triglycerides) were utilized to create an SNE to test their solubilization and nano-emulsification ability. The oil with the highest ability to solubilize SA was chosen due to its significant impact on drug solubilization ability and absorption. The tested oils included Labrafil, Capryol-90, Labrafac, olive oil, anise oil, almond oil, soybean oil, and corn oil. The screened surfactants were hydrophilic non-ionic surfactants (Cremophor El, Tween 20, and Tween 80); they have high HLB values and low oral toxicity. Labrasol, Transcutol, PEG 400, and propylene glycol were the screened cosurfactants. An isothermal method previously reported in the literature [15] was utilized for the solubility determination. In brief, 300 mg of SA was added to 1 mL of each component in screw-capped glass vials and mixed using a vortex mixer. The mixtures were then transferred to a thermodynamic water bath shaker (JULABO™, Julabo Labortechnik GMBH, Seelbach, Germany) for continuous shaking at a speed of 100 rpm at room temperature for three days, followed by centrifugation at 4000 rpm for 25 min. SA concentration was measured spectrophotometrically at 322 nm after the supernatant was diluted with methanol using a magnetic stirrer at 500 rpm for 2 min at room temperature [28]. The results of this study provide important insights into the solubility of SA in various oils, surfactants, and cosurfactants, which is crucial for the formulation of SA in a self-nanoemulsifying dosage form.

### 2.2. Construction of Pseudo-Ternary Phase Diagram

Based on the highest solubility of SA in different components, Labrafil, Cremophor El, and Transcutol were selected as the oil phase, surfactant, and co-surfactant, respectively. Various mixtures with varying concentrations of oil, surfactant, and co-surfactant were prepared, and a pseudo-ternary diagram was plotted using ProSim software (Labège, France, https://www.prosim.net/produit/prosimplus-simulation-optimisation-procede-industriel/ (accessed on 10 June 2023)). Labrafil, Cremophor El, and Transcutol levels varied from 10 to 30% *w*/*w*, 20 to 80 % *w*/*w*, and 10 to 70% *w*/*w*, respectively. All compositions were visually examined for nano-emulsion formation after diluting each mixture 100 times with deionized water [29]. 

### 2.3. Computer-Aided Optimization of SA-SNE Formulation Using Mixture I-Optimal Design

Recently, experimental design has been used in formulation development. This method yields ideal data with the fewest possible experiments and additional information regarding the effect of components on responses [30]. Pre-optimization research determined the proportion of each component (Labrafil, Cremophor El, and Transcutol). These concentrations were optimized using Design Expert (Version 11.1.2.0) software from Stat-Ease, Inc., Minneapolis, MN, USA. Labrafil, Cremophor El, and Transcutol concentrations were established as independent variables or factors. According to the pre-optimization research, the limits of every independent variable were identified and prescribed in the previous section. The sum of all components in a formulation always equals 100 percent. The parameters chosen included transmittance, solubility, and globule size. The optimization batches were chosen following the desirability function. Formulations were selected with a desired factor close to 1.0.

### 2.4. Globule Size, Size Distributions and Zeta Potential

SA-SNEs were diluted with deionized water and subjected to particle size examination. The average particle size, polydispersity index (PDI), and zeta potential were determined using dynamic light scattering on a Malvern Zetasizer (Malvern Instruments, Malvern, UK) [31]. 

### 2.5. Dispersibility Studies

Self-emulsification time was assessed through dispersibility studies. First, 1 mL of SA-SNE was added dropwise to 100 mL of isotonic phosphate buffer (pH 7.4) with gentle agitation using USP Type II (paddle) dissolution apparatus rotating at 50 rpm at 37 ± 0.5 °C. The process of self-emulsification was visually checked. Precipitation was assessed through visual monitoring of the resultant emulsion after 24 h storage at room temperature [14].

### 2.6. Solubility Studies

Solubility studies were conducted by adding excess SA to 1 mL of each SA-SNE mixture. The mixtures were moved to a thermodynamic water bath shaker (JULABO™, Julabo Labortechnik GMBH, Seelbach, Germany) for continuous shaking at a shaking speed of 100 rpm at room temperature for three days, then centrifuged at 5000 rpm for 25 min. The supernatant was diluted with methanol using a magnetic stirrer at 500 rpm for 2 min at room temperature and then subjected to the quantification of SA content spectrophotometrically at 322 nm [32]. 

### 2.7. Transmittance

Using distilled water to reconstitute the SA-SNE, the resulting nano-emulsion was examined visually for any turbidity. A UV-vis spectrophotometer was then used to assess its percent transmittance at 638.2 nm, with distilled water serving as the blank. The experiments were performed following a 100-fold dilution [33].

### 2.8. Differential Scanning Calorimetry Studies

Thermal analysis of SA, Labrafil, Cremophor El, Transcutol, and the optimized SA-SNE mixture was carried out using a differential scanning calorimeter (DSC) (Shimadzu, DSC 60 TSW 60, Kyoto, Japan). The study was conducted within the 50–200 °C range at a scanning rate of 10 °C/min. An empty pan was used as a reference [34].

### 2.9. Transmission Electron Microscopy

A transmission electron microscope (JEOL JEM-1400, Tokyo, Japan) examination of the optimized SA-SNE was performed for visual observation. A copper grid was stained for five minutes at room temperature with a 1 percent *w*/*v* solution of phosphotungstic acid before being treated with a drop of diluted SA-SNE. A transmission electron microscope was used to capture images at a 100 kV accelerated voltage [35].

### 2.10. In Vitro Release Study and Kinetic Analysis 

An in vitro drug release was carried out to compare the release profiles of SA from optimized SA-SNE and its suspension formulation, containing 10 mg of SA each. The in vitro dissolution tests on SA SNE were carried out using the dialysis method. A glass cylinder open at both ends was attached to the shaft of United States Pharmacopoeia (USP) dissolution apparatus (Erweka DT-720, Erweka GmbH, Heusenstamm, Germany), and a dialysis membrane (MWt of 12,000 kDa) was tied at one end of the glass cylinder [31,35]. In vitro drug release tests were conducted in a beaker containing 50 mL of isotonic phosphate buffer (pH 7.4) (dissolution medium). These experiments were conducted at 100 rpm with the dissolving media kept at a constant 37 ± 0.5 °C. At regular time intervals, three milliliters of sample from each formulation were removed, and the same volume of SA-free new isotonic phosphate buffer (pH 7.4) was added instead. At 322 nm, the concentration of SA in each sample was measured spectrophotometrically. The relationship between spectrophotometric absorbance and SA concentration was plotted using the calibration curve. The amount of SA in the media was calculated at various time intervals from the calibration curve. The release kinetics data for sinapic acid suspension were gathered using three different models: Zero Order, First Order, and Diffusion. These models provide insights into the drug release behavior and offer valuable information for the design and formulation of sinapic-acid-based pharmaceutical products.

### 2.11. Pharmacokinetic Study in Rabbits

The pharmacokinetic study was carried out to evaluate the bioavailability of SA from the optimized SA-SNE nebulized dosage form, and it was compared with an oral SA suspension. The ultimate success of any formulation depends on its in vivo performance, so an in vivo study was conducted in a rabbit model. 

Albino New Zealand rabbits obtained from the Nahda University animal center (Beni-Suef, Egypt), of either sex, 3 months old, and weighing between 1.35 and 1.72 kg, were used in this study. They were maintained on a standard pellet diet and water. A total of 12 rabbits were divided into two groups, each containing 6 (*n* = 6) animals. The animals of group A were fed with 1 mL of drug suspension containing 10 mg/Kg of SA by mouth using an oral feeding needle. The animals of group B were given optimized SA-SNE containing the same amount of SA via nebulization using an ultrasonic nebulizer (PARI GmbH, Starnberg, Germany) [36,37].

The ultrasonic nebulizer was configured and connected to a power source for operation. Within the nebulizer, the medication chamber was filled with optimized SA-SNE. Upon activation, the ultrasonic nebulizer emitted high-frequency vibrations (ultrasonic waves) that were transmitted to the liquid medication within the chamber, resulting in the transformation of the medication into a fine mist or aerosol comprising minute medication droplets suspended in the surrounding air. This aerosol was then directed to the rabbits for inhalation, typically facilitated within a suitable chamber, thereby enabling the aerosolized medication to be absorbed directly into the rabbits’ respiratory system, including the lungs and airways [38,39].

The rabbits were anesthetized using diethyl ether, and 0.5 mL of blood was withdrawn from the tip of the ear using a 19–23-gauge butterfly needle away from the base of the ear. Blood flow was stopped by applying pressure with sterile gauze placed at the blood sampling site for approximately 2 min to achieve hemostasis. Blood samples were withdrawn from the marginal ear vein of each rabbit at 0, 1, 2, 3, 4, 6, and 24 h, then collected in 1.5 mL capacity EDTA Eppendorf tubes. The tubes were centrifuged at 5000 rpm for 5 min, and the plasma was separated and stored at −80 °C until analysis. The animal experiments conducted were approved by the Institutional Animal Ethics Committee of Nahda University (Regd. No. NUB-032-022).

### 2.12. Pharmacokinetic Analysis of SA in Plasma through HPLC-UV Method

#### 2.12.1. HPLC Method

HPLC separation was carried out on a stationary-phase ZORBAX Eclipse Plus^®^ C18 column (250 × 4.6 mm, 5 µm), using a mobile phase consisting of acetonitrile and water in the proportion of (98:2, %*v*/*v*) at a flow rate of 1.0 mL/min using ciprofloxacin as an internal standard. The overall run time was 7 min, and 20.0 µL of each sample was injected in triplicates. The UV detection was conducted at 320.0 nm, and the temperature was adjusted to 25 °C.

#### 2.12.2. Sample Preparation

The samples of frozen plasma previously collected from rabbits were thawed to room temperature, 100.0 µL of the collected plasma samples was accurately transferred into a 2 mL centrifuge tube, then 40.0 µL (IS) was added from its stock solution (1.00 mg/mL). The volume was then adjusted to 1.0 mL with acetonitrile. Samples were then vortex-mixed for one minute and centrifuged for 20 min at 4000 rpm; the clear supernatant of each sample was completely taken and evaporated to dryness. The dried samples were then reconstituted with 1.0 mL acetonitrile, and 20.0 µL was injected for analysis.

### 2.13. M^pro^ Enzyme Assay 

Top-scoring compounds were assessed for their in vitro enzyme inhibition activities using a 3CL Protease, tagged (SARS-CoV-2) Assay Kit (Catalogue #: 79955-1, BPS Bioscience, Inc., Allentown, PA, USA) according to the manufacturer’s protocol. The in vitro FRET assay was monitored at an emission wavelength of 460 nm with an excitation at 360 nm using a Flx800 fluorescence spectrophotometer (BioTek Instruments, Winooski, VT, USA).

### 2.14. Antiviral Assay

The antiviral screening was performed using SARS-CoV-2 (wild strain) virus at the Egyptian Company for Production of Vaccines (VACSERA) using strain hCoV-19/Egypt/NRC-3/2020 isolate, which was isolated in the Ministry of Health Laboratories for diagnostic reasons in May 2020. The cytotoxicity and the antiviral activities were investigated using Vero E6 cells (Sigma Aldrich, St. Louis, MO, USA). Then, serial concentrations of the test compounds were incubated with Vero E6 and then infected with SARS-CoV-2 (hCoV-19/Egypt/NRC-3/2020 isolate) at an MOI (multiplicity of infection) of approximately 0.5. DMSO was used as a solvent control. The antiviral screening was performed in a Biological Safety Cabinet Class III BCBS-502 (Biolab Scientific Ltd., Suite 300, Toronto, ON M1V 0B8, Canada) at VACSERA Co., Giza, Egypt. The detailed quantitative real-time PCR antiviral assay results can be found in the Supplementary Materials.

### 2.15. In Silico Study

Docking, molecular dynamics (MD) simulations, and Gaussian accelerated molecular dynamics (GaMD) simulations were carried out according to the previously described methods [40]. The methods are described in detail in the Supplementary Materials.

## 3. Results and Discussion

### 3.1. Screening of Components for SA-SNE Development

SNEDDSs are used to improve the solubility, bioavailability, and therapeutic efficacy of poorly water-soluble drugs [41]. The selection of surfactant, oil, and co-surfactant components can impact the physical properties and performance of the resulting nano-emulsion, such as particle size, stability, and drug release profile [42]. The solubility of pharmaceutical molecules in various components is the most essential requirement for screening components [43]. In this study, a systematic approach was employed to choose these components. Different oils with varying saturation degrees, specifically medium- or long-chain triglycerides, were evaluated to assess their solubilization and nano-emulsification capabilities. The oil demonstrating the highest proficiency in solubilizing SA was selected due to its significant influence on drug solubility and absorption. Additionally, the surfactants chosen for this study, namely Cremophor El, Tween 20, and Tween 80, were carefully screened based on their hydrophilic, non-ionic properties, which are advantageous for promoting emulsification and maintaining stability [25]. These surfactants are known for their high hydrophilic–lipophilic balance values, indicating their suitability for forming stable nano-emulsions [44]. Moreover, their low oral toxicity profile is a crucial consideration to ensure the safety of the delivery system [45]. Furthermore, the preference for oils with medium-length carbon chains over longer-chain oils, such as Labrafil, Capryol-90, and Labrafac, was based on empirical evidence indicating that medium-chain triglycerides exhibit superior solubilization and emulsification capabilities [24]. This choice aligns with the goal of optimizing drug delivery efficiency, underscoring the importance of selecting components that enhance drug solubility and bioavailability. These choices were made to design an efficient self-nanoemulsifying drug delivery system that could improve the solubility and absorption of SA, ultimately enhancing its therapeutic efficacy.

**Figure 1:**
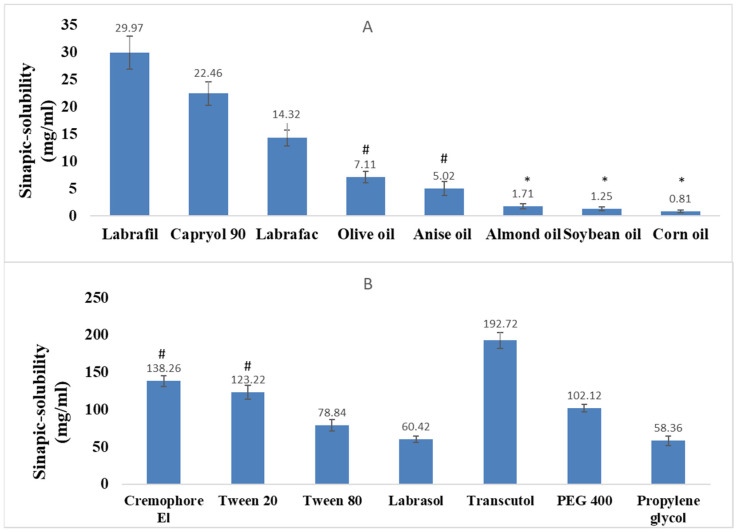
The saturated solubility of sinapic acid in: (**A**) different oils, (**B**) different surfactants and cosurfactants (* and # represent insignificance at *p* < 0.05 using ANOVA and SPSS 16.0 software).

The solubility of SA was determined in various oils, surfactants, and cosurfactants using an isothermal method (Figure 1), and Tukey’s post hoc test and one-way ANOVA were applied to check the significance of the obtained results. Among the oils, Labrafil demonstrated the highest solubilization concentration (29.98 mg/mL) by a significant margin at a P value less than 0.05. It was rated adequate, indicating its potential as an excellent carrier oil for drug delivery applications. Capryol 90 and Labrafac had moderately high concentrations, making them suitable alternatives to Labrafil in various formulations. Olive oil, anise oil, almond oil, soybean oil, and corn oil had lower concentrations, indicating their suitability for use in formulations where low oil concentration is preferred.

Surfactants are used in formulations to improve the solubility and bioavailability of poorly soluble drugs [46]. The results showed that Cremophore El had the highest concentration (138 mg/mL), followed by Tween 20 (122 mg/mL) and Tween 80 (78 mg/mL), while Labrasol had the lowest concentration (60 mg/mL). The choice of surfactant depends on the specific drug and formulation requirements. Cremophore El is often used in intravenous formulations, while Tween 20 and Tween 80 are commonly used in oral formulations due to their low toxicity [47]. Co-surfactants are added to formulations to enhance the solubilization and absorption of drugs. The results indicated that Transcutol had the highest concentration (192 mg/mL) by a significant margin at *p* < 0.05, followed by PEG 400 (102 mg/mL) and propylene glycol (58 mg/mL). The choice of co-surfactant depends on the specific formulation requirements and the compatibility with the surfactant [48]. The differences in the solubility of SA in different oils could be attributed to their physicochemical properties, such as their polarity, molecular weight, and viscosity [49]. The higher the solubility of SA in an oil, the better it can be used as a solvent or carrier for SA in various applications. These findings were consistent with previous studies reporting the high solubility of SA in Labrafil due to its high lipophilicity and low hydrophilicity [15]. Moreover, the high solubility of SA in Cremophor El and Transcutol might be attributed to their ability to form micelles and solubilize hydrophobic drugs [28]. Based on these results, Labrafil, Cremophor EL, and Transcutol, as the oil, surfactant, and cosurfactant, respectively, were further utilized for constructing a ternary phase diagram.

### 3.2. Construction of Pseudo-Ternary Phase Diagram

**Figure 2:**
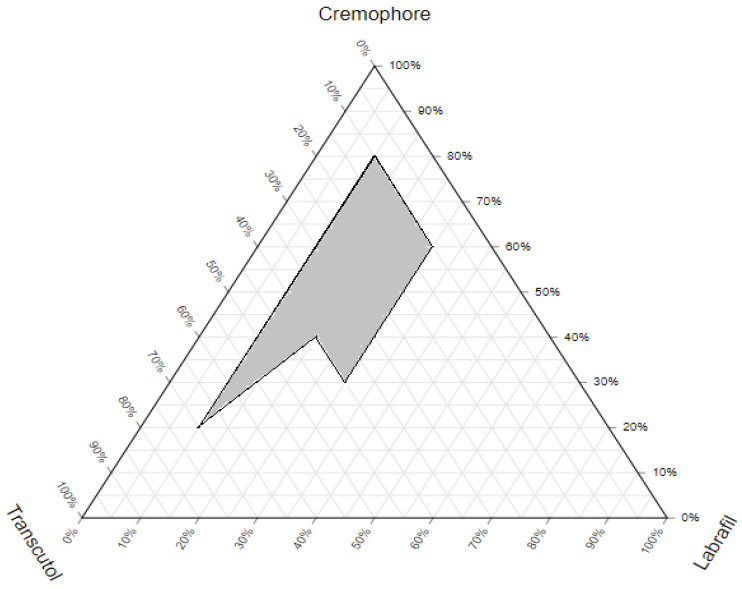
Pseudo-ternary phase diagram created using ProsSim software 1.0 for the selected system (Labrafil as the oil, Cremophor EL as the surfactant, and Transcutol as the co-surfactant.), showing the nano-emulsification area in gray.

A pseudo-ternary phase diagram provided a useful tool for selecting the optimal composition of oil, surfactant, and cosurfactant for the formulation of a stable and efficient SA-SNE. Based on the results obtained from the solubility study, the pseudo-phase diagram was constructed using Labrafil as oil, Cremophor EL as surfactant, and Transcutol as cosurfactant. The ternary phase diagrams (Figure 2) were constructed to determine the concentration range of components required to form a stable nano-emulsion. The darker region in the phase diagram represented the self-emulsification area [50]. It was observed that the addition of surfactant–cosurfactant improved the efficiency of emulsification due to their higher hydrophilicity properties [51]. A stable system was seen with an oil concentration of up to 30% *w*/*w*. The addition of a cosurfactant, Transcutol, was found to improve the self-emulsification property of the system. Furthermore, it was observed that spontaneous emulsion formation was not efficient, with less than 40% *w*/*w* of the surfactant in the system. The results also showed that the drug solubility was influenced by the oil concentration, surfactant, and co-surfactant. As the concentration of the oil increases, the drug solubility decreases. Conversely, as the concentration of the surfactant and co-surfactant increases, the drug solubility increases. This is because the surfactant and co-surfactant help in solubilizing the drug in the microemulsion [52]. The results of this study provided a basis for optimizing the formulation of the SA-SNE.

### 3.3. Computer-Aided Optimization of SA-SNE Formulation Using Mixture I-Optimal Design

Computer-aided optimization of an SA-SNE is an efficient approach to develop an optimal formulation with desirable characteristics. One approach to optimize an SA-SNE formulations is the use of mixture I-optimal design. Following this approach, the formulation variables, such as oil, surfactant, and cosurfactant concentrations, were varied according to a designed experimental plan. The experimental plan was based on a statistical approach that minimized the number of experiments required while providing a comprehensive understanding of the effect of each variable on the response of interest. Computer-aided optimization using mixture I-optimal design had several advantages over traditional experimental approaches [34,53]. It provided a comprehensive understanding of the formulation variables’ effects. This approach also allowed for the optimization of multiple response variables simultaneously, leading to a more efficient formulation process.

The pre-optimization studies helped to identify the ranges of concentrations for the critical components of the SA-SNE formulation, i.e., oil, surfactant, and co-surfactant. To optimize the SA-SNE formulation, the concentrations of oil, SA, and co-surfactant were chosen as independent variables, while globule size, solubility, and percent transmittance were selected as responses. Globule size is an important parameter that determines the physical stability of the SA-SNE [54], while solubility is a critical factor that affects the bioavailability of the drug [31]. Percent transmittance is a measure of the clarity and transparency of the formulation.

A total of 15 formulations were constructed using Design Expert software (Version 11.1.2.0 of Stat-Ease, Inc., Minneapolis, MN, USA) based on the pre-optimization studies. The design matrix of the 15 formulations is presented in Table 1. This design aimed to find the optimal formulation to achieve the desired responses. The design matrix was analyzed using the Design Expert software, and the responses were fitted to a second-order polynomial equation. The model was evaluated using ANOVA, and significant factors were identified.

After developing the models, optimization was conducted using the desirability function. The desirability function combines the responses into a single value between 0 and 1, where 1 is the ideal response. The formulations with the highest desirability factor were selected for further analysis. The R-squared and adjusted R^2^ values provide an indication of how well the model fits the data. In this study, the R-squared and adjusted R^2^ values were relatively close, suggesting that the model fit was adequate. The adequate precision (AP) ratio measures the signal-to-noise ratio and indicates whether the experimental region is well represented by the model. The AP ratios for all responses were greater than 4, which is sufficient for routing the design space. Therefore, the results suggested that the models developed were reliable and suitable for the optimization of the SA-SNE formulation [55].

The results of the design matrix (Table 1) showed that as the percentage of surfactant (X1) increased, the transmittance percentage (Y1) also increased, indicating better transparency of the SA-SNE. However, there was a trade-off between transmittance and particle size (Y3), as the size decreased with increasing surfactant percentage. The co-surfactant percentage (X3) and oil percentage (X2) also affected the solubility (Y2) and size (Y3) of the SA-SNE. The results showed that the transmittance values ranged from 92 to 100%, showing that the samples had good clarity. The solubility values varied from 101.2 to 230.5 mg/mL, which suggested that the SNEDDS effectively enhanced the drug’s solubility. The size of the SA-SNE ranged from 83.6 to 203.1 nm, indicating that the samples were in the nano-emulsion range. The PDI values ranged from 0.134 to 0.310, indicating a low to moderate level of polydispersity. The emulsification time ranged from 35 to 72 s, indicating that the preparation of SA-SNE was relatively easy. The zeta potential values ranged from −17.8 to −12.1 mV, indicating that the samples had a negative charge on their surface.

Table 2 summarizes the regression analysis for the three response variables: globule size, solubility, and % transmittance. The model for all three responses was linear, as indicated by the “Linear” in the “Model” column. In general, the linear model is the most appropriate for the mixture design since the relations between the mixture ingredients are shown by directly proportional responses [56]. In addition, linear models are less complex than other models [57]. The *p*-values for solubility and % transmittance were highly significant at 0.0001 and 0.0185, respectively, indicating that the model terms were significant for these responses. The R-squared values for solubility and % transmittance were high at 0.8931 and 0.4857, respectively, indicating that the model could explain a large proportion of the total variation in these responses. The R-squared value for globule size was lower at 0.5231, indicating that the model explained a lower proportion of the variation in this response. The adjusted R-squared values considered that the number of terms in the model were lower than the R-squared values. However, the adjusted R-squared values for solubility and % transmittance were still relatively high at 0.8753 and 0.4000, respectively, indicating a good model fit. 

The adjusted R-squared value for globule size was lower at 0.4436, indicating that the model might be overfitting the data. The predicted R-squared values were lower than the R-squared values, indicating that the model could have limited predictive ability for new observations. The adequate precision ratios for all three responses were above the recommended threshold of 4, indicating that the model could navigate the design space [58]. The coefficients of variation (%CV) for solubility and % transmittance were low, indicating good precision and reproducibility of the measurements. The %CV for globule size was higher, indicating more variability in the measurements. The PRESS values for all three responses were relatively high, indicating that the model might not fit the data and could lead to residual error. This suggested that the model could be further improved by including additional terms or refining the experimental design.

The regression equations (Table 2) represent mathematical models that predicted the response of the dependent variable, Y, based on the values of the independent variables, X1, X2, and X3. The dependent variable could be a drug delivery system’s physical properties, such as viscosity, surface tension, or solubility. In contrast, the independent variables were the compositions of the system, represented by the amounts of Cremophor EL (X1), Labrafil (X2), and Transcutol (X3). The general form of the equations is Y = aX1 + bX2 + cX3, where a, b, and c are coefficients that represent the contribution of each independent variable to the dependent variable. The specific values of these coefficients were obtained through statistical analysis of experimental data, such as regression analysis, and were unique to the specific system and dependent variable studied. 

From the regression data of the linear models, the equations mentioned in Table 2 were obtained. These equations showed positive values for the three independent variables, showing their positive effect on the three responses. In Equation (1), the Y value was predicted based on the specific coefficients of 136.49 for X1 (Cremophor EL), 239.65 for X2 (Labrafil), and 6.38 for X3 (Transcutol). In Equation (2), the coefficients were 81.77 for X1, 221.32 for X2, and 160.34 for X3, and in Equation (3), the coefficients were 100.49 for X1, 91.56 for X2, and 95.35 for X3. These equations could be used to predict the response of the dependent variable, such as the solubility of a drug, based on the independent variables, such as the amounts of surfactant, oil, and co-surfactant in a self-nanoemulsifying drug delivery system. The equations could also be used to optimize the system’s composition to achieve a desired response or to identify the independent variables with the most influence on the dependent variable. Equations (1) and (2) had a similar range of coefficients for X1 and X2, but Equation (2) had a much higher coefficient for X3. This suggested that X3 (Transcutol) might be more critical in predicting the response in Equation (2) than in Equation (1). Equation (3) had lower coefficients overall compared to Equations (1) and (2), which suggested that the independent variables might have a minor impact on the dependent variable in this case.

**Figure 3:**
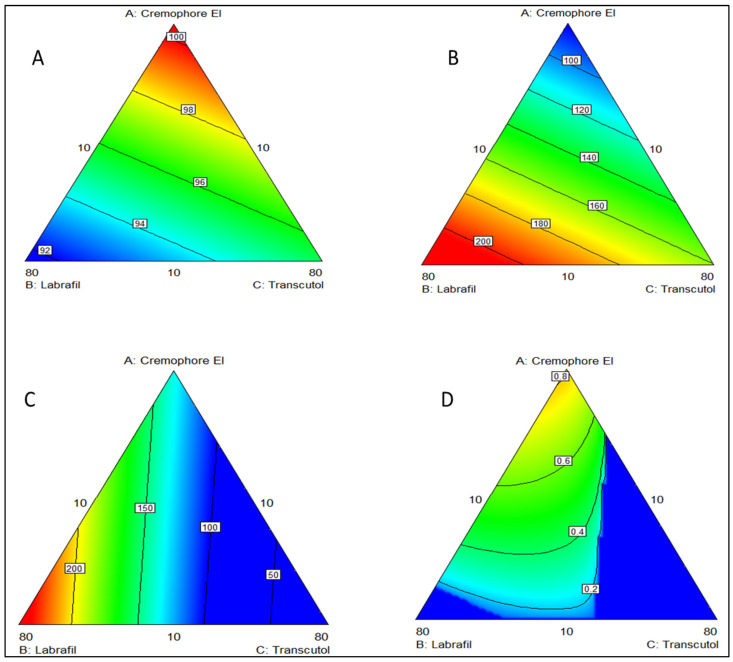
2D contour plots for the effect of the independent variables (concentration of oil, surfactant, and cosurfactant) on (**A**) enhancing the % of transmittance up to 100%, (**B**) lowering the globule size from 200 to 100 nm, (**C**) enhancing the solubility, and (**D**) increasing the desirability.

**Figure 4:**
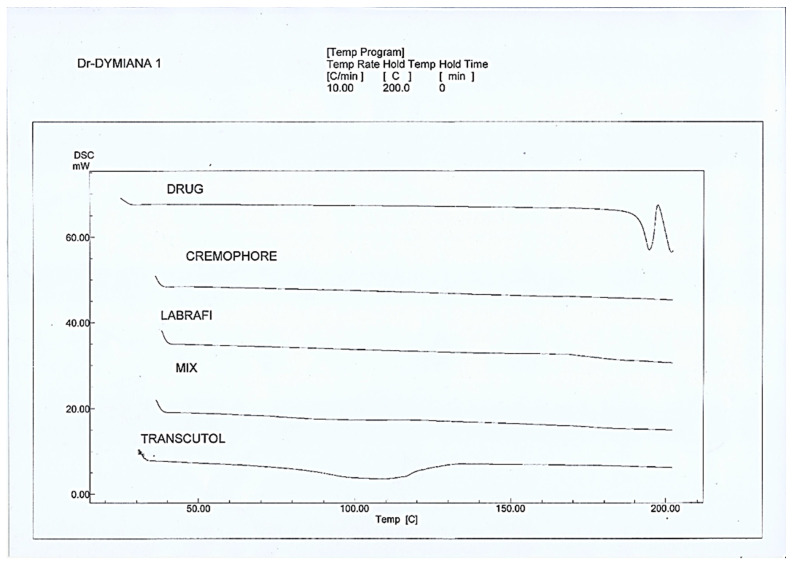
DSC thermograms of sinapic acid (drug), Cremophore El, Transcutol, Labrafil, and the optimum SNEDDS mix.

**Figure 5:**
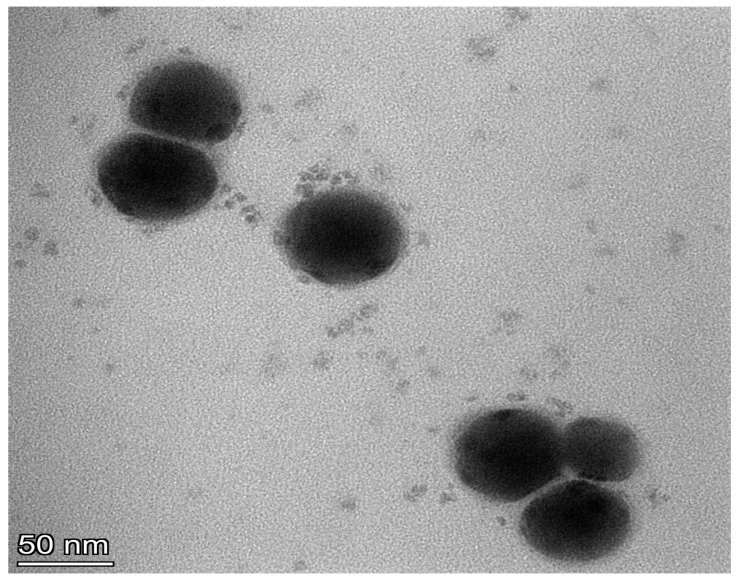
Photomicrograph of the optimum SNE, taken using TEM (50,000×).

**Figure 6:**
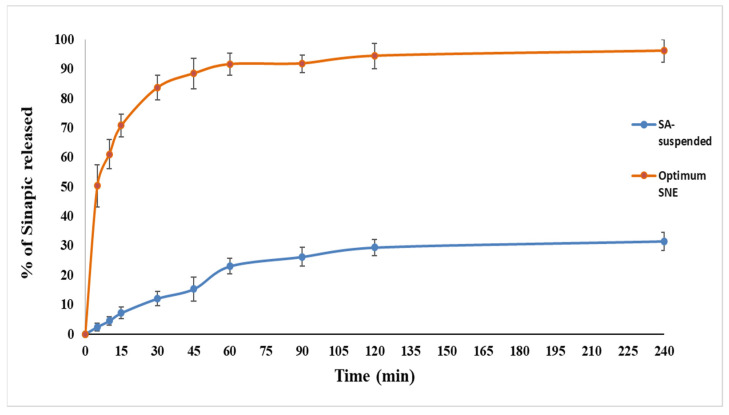
In vitro release of sinapic acid from the optimized SNE compared with the sinapic suspension.

Figure 3, Figure 4, Figure 5 and Figure 6 illustrate model equations and show the effects of independent variables on responses. They were created to interpret the mixture region. In the response surface, each factor (pure mixture component) is represented in a corner of an equilateral triangle; each point within this triangle refers to a different proportion of components in the mixture. The maximum percentage of each ingredient considered in the regression is placed in the corresponding corner, while the minimum is positioned in the middle of the opposite side of the triangle. The 2D contour plots show the effect of the independent variables, surfactant percentage (X1), oil percentage (X2), and co-surfactant percentage (X3), on the % of transmittance (Y1) (Figure 3A). The contour plot indicates that the % of transmittance was primarily influenced by the surfactant percentage (X1) and oil percentage (X2). The co-surfactant percentage (X3) had a relatively weaker effect on the transmittance. The contour lines on the plot are elliptical, which suggests an interactive effect of the two variables on the transmittance. The maximum % of transmittance is observed at the center of the plot, which corresponds to the formulation with a higher surfactant percentage and a lower oil percentage. Based on the contour plot, it could be inferred that a surfactant percentage of around 40–50% and an oil percentage of around 10–20% could provide optimum transmittance for the formulation. The contour plot provided a graphical representation of the relationship between the independent and response variables and helped identify the optimum formulation conditions.

The effect of the independent variables on the globule size Figure 3B reveals that the surfactant percentage and oil percentage had opposite effects on the globule size. As the surfactant percentage increased, the globule size decreased, while increasing the oil percentage resulted in an increase in globule size. This trend is consistent with emulsions’ general behavior, where surfactants stabilize oil droplets, preventing them from coalescing into larger droplets [59]. In addition to the surfactant and oil percentages, the contour plot also reveals that the lowest values of globule size occurred when the surfactant percentage was high (above 40%) and the oil percentage was low (below 20%). This result indicated that the choice of surfactant and oil types, as well as their relative concentrations, could have a significant impact on the globule size of an emulsion. The contour plot could also be used to estimate the optimal values of the independent variables that resulted in the desired globule size. For example, if the target globule size was around 100 nm, the contour plot suggests that this could be achieved by using a high surfactant percentage (above 40%) and a low oil percentage (below 20%).

Figure 3C illustrates the effect of the independent variables on SA solubility. The contour plot shows that the co-surfactant percentage had a strong positive correlation with SA solubility. As the co-surfactant percentage increased, SA solubility also increased. This trend is depicted by the contour lines that move upwards and to the right, indicating higher solubility values. Therefore, it could be inferred that a higher co-surfactant percentage might help improve the solubility of SA. However, the effect of emulsification time on SA solubility was less clear. There was a slight trend of increasing solubility with increasing emulsification time, but the effect was not as strong as the effect of co-surfactant percentage. The contour lines for emulsification time are more horizontal, indicating that the effect of this variable on SA solubility was less significant compared to the co-surfactant percentage.

Based on the regression models, optimal factor settings were selected using Design-Expert^®^ in order to identify experimental settings in which all desirability criteria were met as much as possible. The optimization process of the design was based on minimizing globule size and maximizing both the transmittance and solubility. According to the optimization parameter input, the software generated an optimum formulation (F 16), which was produced and analyzed. The composition of this optimized formula is described in Table 3. The effect of formulation factors on desirability is shown in Figure 3D. The optimized formula had a droplet size of 83.6 nm with a small PDI OF 0.164, SA solubility of 127.1 ± 3.3, and transmittance of 100%. Experimental and predicted values of the optimum SNE (F 16) were compared and showed close resemblance with a small relative prediction error (less than 9%), suggesting the validity of the generated regression equations. 

### 3.4. Differential Scanning Calorimetry Studies

Thermograms of SA, Labrafil, Cremophor El, Transcutol, and optimized SA-SNE physical mixture were obtained using a differential scanning calorimeter, as shown in Figure 4. In this figure, each curve represents a different material. The figure allowed us to observe each material’s melting point and other thermal properties, which are important parameters that affect the stability and bioavailability of drugs. The sharp endothermic peaks observed for SA, Cremophore El, and Transcutol at around 150 °C, 60 °C, and 50 °C, respectively, indicate that these materials had high melting points. This means it might be more difficult for these to be dispersed in water and absorbed by the body.

In contrast, Labrafil and the optimized SA-SNE mix showed broad endothermic peaks at around 40–50 °C, indicating their lower melting points. These materials are more suitable for drug delivery systems as they can be easily dispersed in water and absorbed by the body [31,60,61]. The change in the melting behavior of SA could be attributed to the inhibition of its crystallization and solubilization of SA in the SA-SNE. Therefore, it could be concluded that SA in the SA-SNE was amorphous [62]. It is known that transforming the physical state of a drug to the amorphous or partially amorphous state leads to a high-energy state and high disorder, resulting in enhanced solubility [63,64].

### 3.5. Transmission Electron Microscopy

TEM photographs of the SA-SNE formulae after dilution with distilled water are shown in Figure 5 and were interpreted for surface morphology and globule size. From the presented figures, it is apparent that globules of all formulae were well dispersed, and no globule aggregation occurred. TEM analysis revealed that most formulae showed spherical and homogeneous droplets with a small size less than 100 nm, which satisfies the criteria of the nanometric size range required for the nanoemulsifying formula. These results support the results obtained through dynamic light scattering for the optimized formula, which had a droplet size of 83.6 nm with a small PDI OF 0.164.

### 3.6. In Vitro Release 

In vitro release profiles of the SA-SNE optimized formulation were compared with the SA suspension. A rapid initial release of SA from the SA-SNE was observed. As shown in Figure 6, the SA suspension released less than 25% within 24 min, with much lower drug release than the optimized-formula SA-SNE, which released more than 92% of SA simultaneously. The highest release of SA from the formulation SA-SNE was possible due to its lowest droplet size (83.6 nm), lowest PDI value (0.164), and high Cremophore El concentration (80%). The higher drug release rate from the formula is because the quantitative drug release from developed nano-emulsions is droplet-size-dependent. This led to a greater interfacial area in the nano-emulsion with small drops, promoting rapid drug release [53]. These results could be explained by the fact that the SA-SNE was composed of a mixture of oil, surfactant, and co-surfactant, which spontaneously form nano-emulsions when exposed to an aqueous environment. The nano-emulsion droplets’ small size increases the drug’s surface area in contact with the dissolution medium, which enhances the SA dissolution and release rate [65]. Additionally, the surfactant and co-surfactant components of the SA-SNE can help solubilize the SA and keep it in solution, further enhancing the release rate [66,67]. In contrast, an SA suspension was composed of solid SA particles suspended in a liquid medium. The kinetics analysis of the release data began by assessing the goodness of fit for each model, as indicated by the R-squared (R^2^) values (Table 4). The R^2^ value quantifies how well each model aligns with the experimental data. It was observed that the Diffusion model exhibited the highest R^2^ value of 0.9226, signifying an excellent fit to the release kinetics of sinapic acid. This result suggests that the Diffusion model is particularly well suited for describing the release behavior of the drug from the suspension. In the Diffusion model for the suspension, a steep negative slope (−2.4448) was observed, indicating a rapid release rate of sinapic acid. This suggests that sinapic acid is released quickly from the suspension. Conversely, in the First Order model for the nano-emulsion, the negative slope (−0.0051) suggests a gradual decrease in sinapic acid concentration over time. 

The release rate of the SA from the suspension was dependent on the rate of dissolution of the SA particles in the liquid medium. Since SA is poorly soluble [68], the dissolution rate from the suspension might be slow, resulting in a lower release rate than the SA-SNE. In summary, when comparing the release kinetics of sinapic acid in the suspension and the self-nanoemulsifying drug delivery system, it is evident that the nano-emulsion formulation offers several advantages. The nano-emulsion exhibited a good fit, statistical significance, and lower variability in parameter estimation. The gradual release profile observed in the nano-emulsion suggests enhanced solubility and controlled release behavior compared to the rapid release from the suspension. These findings underscore the efficacy of the nanoemulsifying system in improving the solubility and release kinetics of sinapic acid, making it a promising approach for pharmaceutical product development.

### 3.7. Pharmacokinetic Analysis of SA in Plasma through HPLC-UV Method

**Figure 7:**
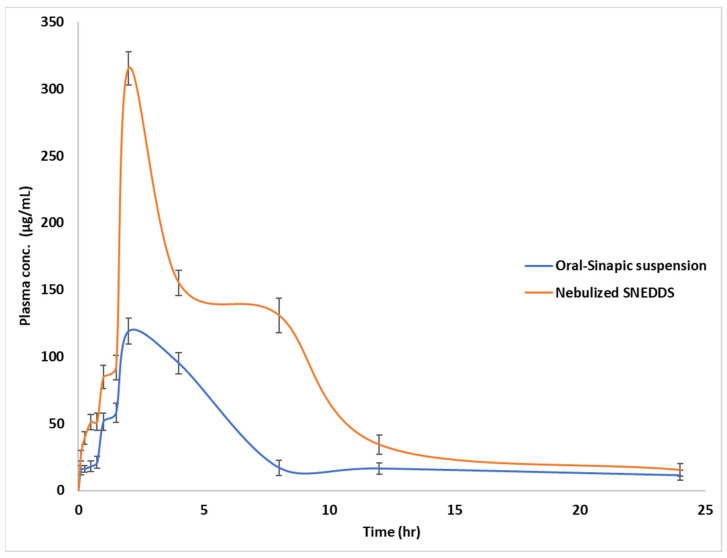
Mean sinapic acid plasma concentration–time curves following administration of oral sinapic acid suspension and nebulized SNE optimized formula to Wistar albino rabbits (n = 6) at (*p* < 0.05).

The pharmacokinetic parameters for SA in the oral pure SA suspension and the nebulized optimum SA-SNE are presented in Table 5. These parameters provide information on the body’s drug absorption, distribution, metabolism, and excretion. The results show that the nebulized optimized SA-SNE formulation resulted in higher C_max_ (maximum plasma concentration) by 2.65 times and AUC_0–24_ (area under the plasma concentration–time curve from 0 to 24 h) by 2.43 times compared to the oral SA suspension, Figure 7. This suggests that the optimized SA-SNE formulation improved the bioavailability of SA by enhancing its absorption and reducing its first-pass metabolism. The higher C_max_ also indicates that the drug reached its peak concentration faster in the blood with the optimized SA-SNE formulation, as shown by the similar T_max_ (time to reach C_max_) values for both formulations. The shorter half-life (t_1/2_) and mean residence time (MRT) of SA with the optimized SA-SNE formulation suggests that the drug was eliminated from the body more quickly compared to the oral suspension [69,70]. This could be due to the increased absorption of SA in the optimized SA-SNE formulation, which could result in faster clearance and metabolism of the drug. Overall, the results of this study suggest that the nebulized optimized SA-SNE formulation could improve the pharmacokinetic profile of SA, which could have implications for its clinical use as a therapeutic agent.

### 3.8. In Vitro Antiviral Activity Study

The IC_50_ for SA’s in vitro antiviral activity against SARS-CoV-2 was determined to be 2.53 ± 0.065 μg/mL. This value is considered 64-fold higher than the reference antiviral drug remdesivir (IC_50_ = 0.036 ± 0.002 μg/mL). The CC_50_ values for SA and the reference antiviral medication remdesivir against the host cells were very low (196.23 ± 15.1 and 170.8 ± 9.8 μg/mL, respectively). It is worth noting that the SA-free SNEDDS did not show any cytotoxicity up to 300 μg/mL (CC_50_ > 300 μg/mL).

Compared to the unformulated SA, the newly prepared nano-formulation significantly decreased viral multiplication (IC_50_ = 0.095 ± 0.0005 μg/mL). Accordingly, the new formulation’s antiviral efficacy became comparable to remdesivir’s. This significant bioactivity improvement might be a direct result of the enhanced cellular delivery of SA.

### 3.9. In Vitro M^pro^ Inhibitory Activity

**Figure 8:**
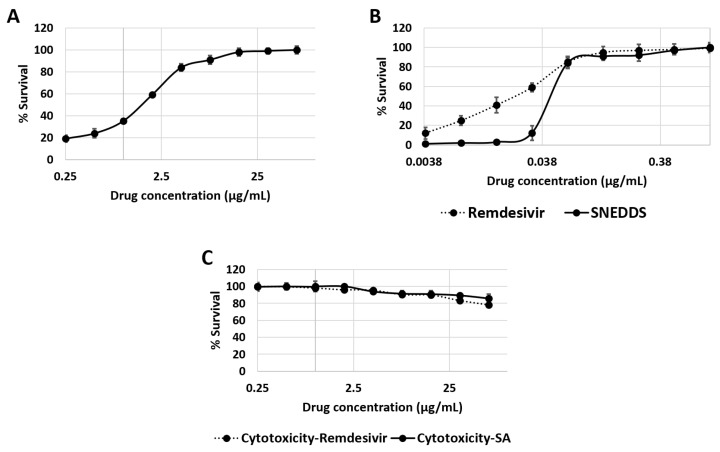
Antiviral activity of SA and its nano-formulation (i.e., SNEDDS) ((**A**,**B**), respectively) against SARS-CoV-2. The antiviral activity of SNEDDS was compared to that of remdesivir in the same Figure (**B**). Cellular cytotoxicities of both SA and remdesivir against Vero E6 cells (**C**). The assays were carried out in triplicate (n = 3).

In the present study, we tested SA for its inhibitory activity against the viral M^pro^. The reason for selecting this specific target for testing was the recently published study about baicalein that proved the ability of this natural product to inhibit M^pro^ activity [71]. As shown in Figure 8, the baicalin scaffold is significantly close to SA, with a Tanimoto similarity index of 0.46. The key difference between the two structures is that SA has a terminal hydrophilic carboxylate moiety, while the reference inhibitor baicalin has a hydrophobic benzene ring. Accordingly, both compounds showed comparable in vitro M^pro^ inhibitory activity (IC_50_ = 6.74 ± 0.95 and 1.12 ± 0.35 μM, respectively) [71].

### 3.10. Investigation of SA’s Mode of Interaction with SARS CoV-2 M^pro^

Being an M^pro^ inhibitor, SA’s modeled structure was docked into the enzyme active site crystal structure (PDB ID: 6M2N). The resulting poses (10 poses) were of convergent orientations, where the RMSD between the pose of the best score and that of the worst one was 1.79 Å. Accordingly, we chose the best-scoring pose for the subsequent investigations.

**Figure 9:**
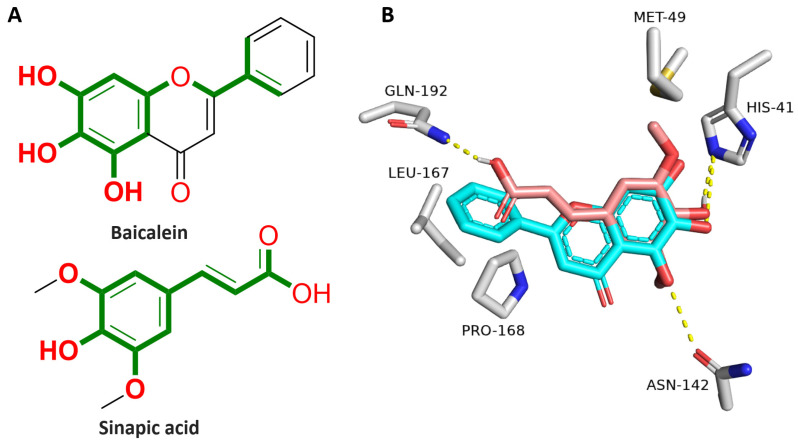
(**A**) Chemical structures of both baicalein and SA showing the structural similarity in green. (**B**) Binding modes of both baicalein and SA in alignment with each other inside the M^pro^ active site. The green color indicates the structural similarity between both molecules.

As shown in Figure 9, both SA and baicalein structures were aligned inside the M^pro^ active site, where they shared some interactions, particularly the hydrophilic ones (e.g., H-bonds with HIS-41 and ASN-142). The main difference between the interactions of each structure was that the carboxylate moiety of SA was involved in H-binding with GLN-192. In contrast, baicalein’s extended aromatic ring (i.e., ring B) was involved in hydrophobic interactions with LEU-167 and PRO-168. 

Accordingly, we tried to validate the SA interaction inside the M^pro^ active site by (i) calculating the absolute binding free energy (Δ*G*_Binding_) of SA; (ii) running 100 ns long molecular dynamics (MD) simulations to study the degree of SA stability inside the M^pro^ active site; and (iii) running Gaussian accelerated molecular dynamics (GaMD) simulations to reveal the binding pathway of SA to the M^pro^ active site.

**Figure 10:**
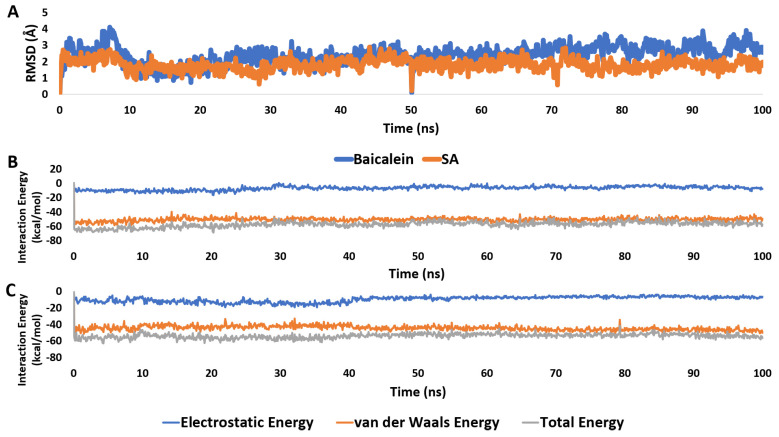
(**A**): RMSDs of SA and baicalein structures inside the M^pro^ active site throughout 100 ns long MD simulation. (**B**,**C**) Interaction energies (i.e., electrostatic and van der Waals energies) of SA and baicalein structures inside the M^pro^ active site over the course of 100 ns long MD simulation.

First, the docking poses of SA and baicalein were subjected to 100 ns long MD simulations. As shown in Figure 10, both structures achieved stable binding throughout MD simulation, with an average RMSD of 2.23 Å and 2.16 Å for baicalein and SA, respectively. Hence, their calculated interaction energies in terms of electrostatic and van der Waals energies were convergent (−66.56 and −61.45 kcal/mol, respectively). In addition, their calculated absolute binding free energies (Δ*G*_Binding_) were also convergent (−9.36 and −8.78 kcal/mol, respectively).

**Figure 11:**
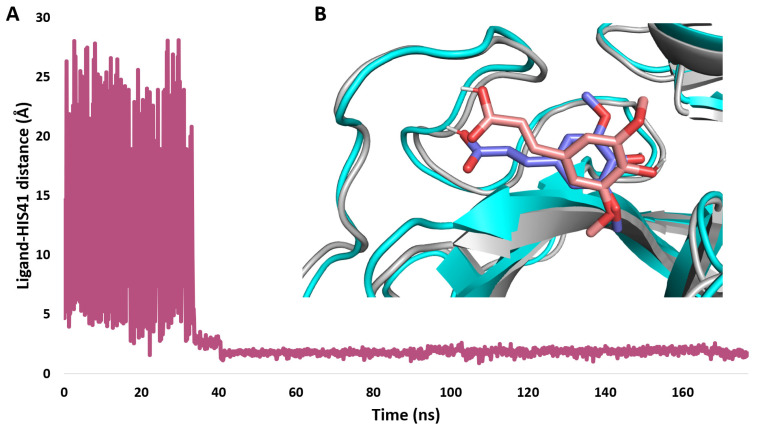
(**A**): Simulation of SA binding event inside the M^pro^ active site observed in GaMD simulation after ~40 ns. (**B**): Structure alignment of the SA docking pose (blue structure) on its most populated structure after binding inside the active site of M^pro^ during the GaMD simulation (brick-red structure). RMSD value between the two states = 2.61 Å. A video showing the binding event of SA inside the active site of M^pro^ can be found on the Zenodo website: https://zenodo.org/record/8175715 (accessed on 10 June 2023).

Second, we tried to re-produce the docking pose of SA inside the M^pro^ active site by running five independent GaMD simulations. Five copies of the SA structure were simulated for 200 ns each, with each copy located at least 20Å away from the modeled M^pro^ structure in the solvent box. As shown in Figure 11, ligand binding was observed in one of the five simulations after ~40 ns and remained stable in the M^pro^ active site until the completion of the simulation. The binding state (Figure 10B) was highly similar to the docking pose chosen earlier (RMSD = 2.61 Å), suggesting this is the most likely binding pose. 

These results indicate that SA exhibited a binding profile comparable to that of the co-crystalized inhibitor baicalein.

## 4. Conclusions

In 2021, we proved that sinapic acid (SA) selectively inhibited SARS-CoV-2 viral replication in vitro by targeting its envelope protein. However, it lacked druggability properties, with low water solubility and poor pharmacokinetic properties. This study successfully developed and optimized a self-nanoemulsifying drug delivery system (SNE) for SA to overcome its poor solubility and enhance its therapeutic efficacy. Our in-depth screening identified Labrafil, Cremophor EL, and Transcutol as suitable choices for the formulation. The construction of a pseudo-ternary phase diagram provided valuable insights into the optimal composition of the SA-SNE formulation, ensuring stability and efficient emulsification. Mixture I-optimal design facilitated computer-aided optimization, considering key factors such as globule size, solubility, and transmittance. The optimized SA-SNE formulation exhibited promising characteristics, including small globule size, high solubility, and rapid drug release. These properties are crucial for improving the bioavailability of SA. Pharmacokinetic analysis revealed that the optimized SA-SNE formulation via nebulization resulted in higher maximum plasma concentration (C_max_) and area under the plasma concentration–time curve (AUC_0–24_) compared to the oral SA suspension. This indicated enhanced absorption and reduced first-pass metabolism of SA. Furthermore, the SA-SNE formulation demonstrated potent antiviral activity against SARS-CoV-2, with an IC_50_ comparable to the reference antiviral drug remdesivir. In silico, modeling, and Gaussian accelerated molecular dynamics (*G*aMD) experiments demonstrated that SA could interact with and inhibit the viral main protease (M^pro^). This suggests the potential of the SA-SNE formulation as a promising candidate for antiviral therapy with multiple modes of action.

## Figures and Tables

**Table 1:** The dependent and independent variables for the prepared SNEDDS using a mixture of experimental designs.

Sample No.	Surfactant (%) (X1)	Co-Surfactant (%) (X3)	Oil (%) (X2)	Transmittance % (Y1)	Solubility (mg/mL) (Y2)	Size nm (Y3)	PDI	Emulsification Time (s)	Zeta Potential (mV)
1	20	70	10	94	230.5 ± 5.2	191.2	0.214	41	−17.1
2	30	60	10	93	210.2 ± 7.3	184.4	0.291	48	−13.3
3	40	50	10	92	199.4 ± 4.6	203.1	0.232	55	−14.6
4	40	40	20	100	174.3 ± 3.1	94.93	0.176	67	−14.2
5	40	30	30	100	128.6 ± 1.4	102.4	0.137	75	−17.8
6	50	40	10	95	153.5 ± 3.6	189.5	0.162	46	−16.5
7	50	30	20	100	134.2 ± 4.2	95.35	0.219	45	−12.1
8	50	20	30	94	117.3 ± 2.3	169.5	0.241	64	−12.4
9	30	40	30	93	129.8 ± 1.9	195.3	0.263	72	−14.7
10	60	30	10	100	184.2 ± 3.7	93.7	0.231	43	−16.9
11	60	20	20	100	156.4 ± 6.2	97.4	0.156	46	−15.7
12	60	10	30	99	101.2 ± 2.7	125.6	0.175	61	−16.1
13	70	20	10	100	118.7 ± 3.1	88.8	0.310	35	−13.4
14	70	10	20	100	111.9 ± 4.2	95.3	0.134	37	−14.6
16	80	10	10	100	127.1 ± 3.3	83.6	0.164	42	−15.1

**Table 2:** Data of regression analysis for responses.

	Globule Size (Y1)	Solubility (Y2)	% Transmittance (Y3)
Model	Linear	Linear	Linear
*p*-value	0.0118	0.0001	0.0185
R-squared	0.5231	0.8931	0.4857
Adjusted R^2^	0.4436	0.8753	0.4000
Predicted R^2^	0.3114	0.8470	0.2459
Adequate precision	7.523	20.140	6.990
%CV	26.53	9.18	2.52
PRESS	21,902.93	3339.41	105.38
Y1 = +136.49 X1 + 239.65 X2 + 6.38 X3			(Equation (1))
Y2 = +81.77 X1 + 221.32 X2 + 160.34 X3			(Equation (2))
Y3 = +100.49 X1 + 91.56 X2 + 95.35 X3			(Equation (3))

**Table 3:** Composition of the optimized SNE system.

Surfactant	Oil	Co-Surfactant
Cremophore El	Labrafil	Transcutol
80%	10%	10%

**Table 4:** Kinetics analysis of the release data.

		Model	
	Zero	First	Diffusion
Slope	−0.13688	−0.00073	−2.44477
Intercept	93.24012	1.969396	100.7459
R^2^	0.745386	0.773053	0.922613
dF	8	8	8
F	23.42007	27.25046	95.37716
SE slope	0.028285	0.000139	0.250331
SE int	2.637903	0.012988	1.963146
SE y	6.270964	0.030876	3.457209
SS reg	920.9942	0.025979	1139.976
SS resid	314.5999	0.007627	95.61835
C.V.%	7.393113	1.604223	53.07682

**Table 5:** Pharmacokinetic parameters for sinapic acid following administration of oral pure sinapic acid suspension and the nebulized optimized SNEDDS.

Parameters	Oral Sinapic Acid Suspension	Optimized SNEDDS
C_max_ (µg/mL)	119.02 ± 13.2	315.31 ± 20.3
T_max_ (h)	2	2
AUC_0–24_ (µg·h/mL)	766.48 ± 130	1865.17 ± 185
t_1/2_ (h)	8.38 ± 0.62	5.76 ± 0.35
AUC_0–∞_ (µg·h/mL)	904.84 ± 27.2	1993.57 ± 43.2
MRT_0–∞_ (h)	11.36 ± 1.8	8.34 ± 1.4

## Data Availability

The data presented in this study are available in this article and Supplementary Materials. All references related to the experimental data in the SI are listed below [72,73,74,75,76,77,78,79,80,81,82].

## Supplementary Materials

The following supporting information can be downloaded at: https://www.mdpi.com/article/10.3390/pharmaceutics15112531/s1, Figure S1: Initial model used in the GaMD simulations. Sinapic acid is shown as green spheres and M^pro^ as cyan ribbons. Golden-yellow balls represent the Na^+^ and Cl^−^ ions.
