# Supplementary material for: Development and Evaluation of a Self-Nanoemulsifying Drug Delivery System for Sinapic Acid with Improved Antiviral Efficacy against SARS-CoV-2
Source: Pharmaceutics. 2023 Oct 25;15(11):2531. doi: 10.3390/pharmaceutics15112531 (PMC10674535; doi:10.3390/pharmaceutics15112531)
Supplement: Supplementary file 1 [file pharmaceutics-15-02531-s001.zip › pharmaceutics-2561328-supplementary.pdf]

Supporting Information

Development and Evaluation of a Self-Nanoemulsifying Drug Delivery System for Sinapic Acid with Improved Antiviral Efficacy against SARS-CoV-2

1. Methods

1.1 Molecular dynamics simulation (MDS)

For performing MDS experiments, the Desmond v. 2.2 software was used [1, 2], which applies the OPLS-2005 force field [3]. Protein systems were built using the System Builder option, where the protein structure was checked for any missing hydrogens, the protonation states of the amino acid residues were set (pH = 7.4), and the co-crystallized water molecules were removed. Thereafter, the whole structure was embedded in an orthorhombic box of TIP3P water together with 0.15 M Na^+ and Cl^- ions in 20 $Å^3$ solvent buffer. The prepared systems were then energy minimized and equilibrated for 10 ns. For protein-ligand complexes, the top-scoring poses were used as a starting point for simulation. The Desmond software automatically parameterizes inputted ligands during the system building step according to the OPLS force field. For simulations performed by NAMD 3.0 [4], the protein structures were built and optimized by using the QwikMD toolkit of the VMD software. The parameters and topologies of the compounds were calculated using the Charmm27 force field with the online software Ligand Reader and Modeler (http://www.charmm-gui. org/?doc=input/ligandrm, accessed on 27 April 2022) [5]. The generated parameters and topology files were loaded to VMD to readily read the protein-ligand complexes without errors and then conduct the simulation step.

1.2 Absolute binding free energy calculation

Binding free energy (ΔG) calculations were performed using the free energy perturbation (FEP) method [5], which has been described in detail in a recent article by Kim and coworkers [5]. The method calculates the binding free energy $\Delta G_{\text{binding}}$ according to the following equation: $\Delta G_{\text{binding}} = \Delta G_{\text{Complex}} - \Delta G_{\text{Ligand}}$. The value of each ΔG is estimated from a separate simulation using NAMD 3.0 software. All input files required for simulation by NAMD 3.0 can be prepared by using the website Charmm-GUI (https://charmm-gui.org/?doc=input/afes.abinding). These files can then be used in NAMD to produce the required simulations applying the FEP calculation function in NAMD. The equilibration (5 ns long) was achieved in the NPT ensemble at 310 K and 1 atm (1.01325 bar) with the Langevin piston pressure (for "Complex" and "Ligand") in the presence of the TIP3P water model. Then, 10-ns FEP simulations were performed for each compound, and the last 5 ns of the free energy values were measured for the final free energy values [5]. Finally, the generated trajectories were visualized and analyzed using the VMD software. In their recent benchmarking study Ngo and co-workers found that the FEP method of determining ΔG was the most accurate one in predicting M^{pro} inhibitors [6].

1.3 Gaussian accelerated molecular dynamics (GaMD) simulation

GaMD simulations were performed starting from the X-ray structure of the M^{pro} of SARS-CoV-2 (PDB ID: 7LTJ; accessed on 15 April 2022). The co-crystallized inhibitor was removed during the setup of the simulations. Ligand parameters for the structure of korupensamine A (**1a**) were assigned according to the Charmm27 force field [5]. The Charmm27 force forcefield [5] parameters were used for all receptor residues. Simulations were performed using NAMD 3.0 [4]. Each system was first energy minimized using the steepest descent and conjugate gradient methods. Then it was gradually heated with the Langevin thermostat to 310 K over 30 ps at constant volume using 1-fs time steps. Initial velocities were sampled from the Boltzmann distribution while keeping weak restraints on the solute and the ligand. Each system was then equilibrated in the isothermal-isobaric ensemble (NPT), at 310 K, using constant pressure periodic boundary with an average

pressure of 1 atm. Isotropic position scaling was used to maintain the pressure with a relaxation time of 2 ps. Non-bonded interactions were cut off at 8.0 Å, and long-range electrostatic interactions were computed using the particle mesh Ewald (PME) [4]. The Secure Hash Algorithm Keccak SHAKE algorithm was used to keep bonds involving H atoms at their equilibrium length. For the integration of Newton's equations, a 2-fs time step was utilized. Simulations started with 10-ns short classical molecular dynamics simulation used to collect potential statistics for calculating the GaMD acceleration parameters followed by 50-ns equilibration after adding the boost potential, and finally multiple independent GaMD production runs with randomized initial atomic velocities. All GaMD simulations were run at a "dual boost": One boost potential was applied to the dihedral energetic term and another to the total potential energetic term. VMD 1.9.3 [4] was used to analyze the GaMD simulation trajectories.

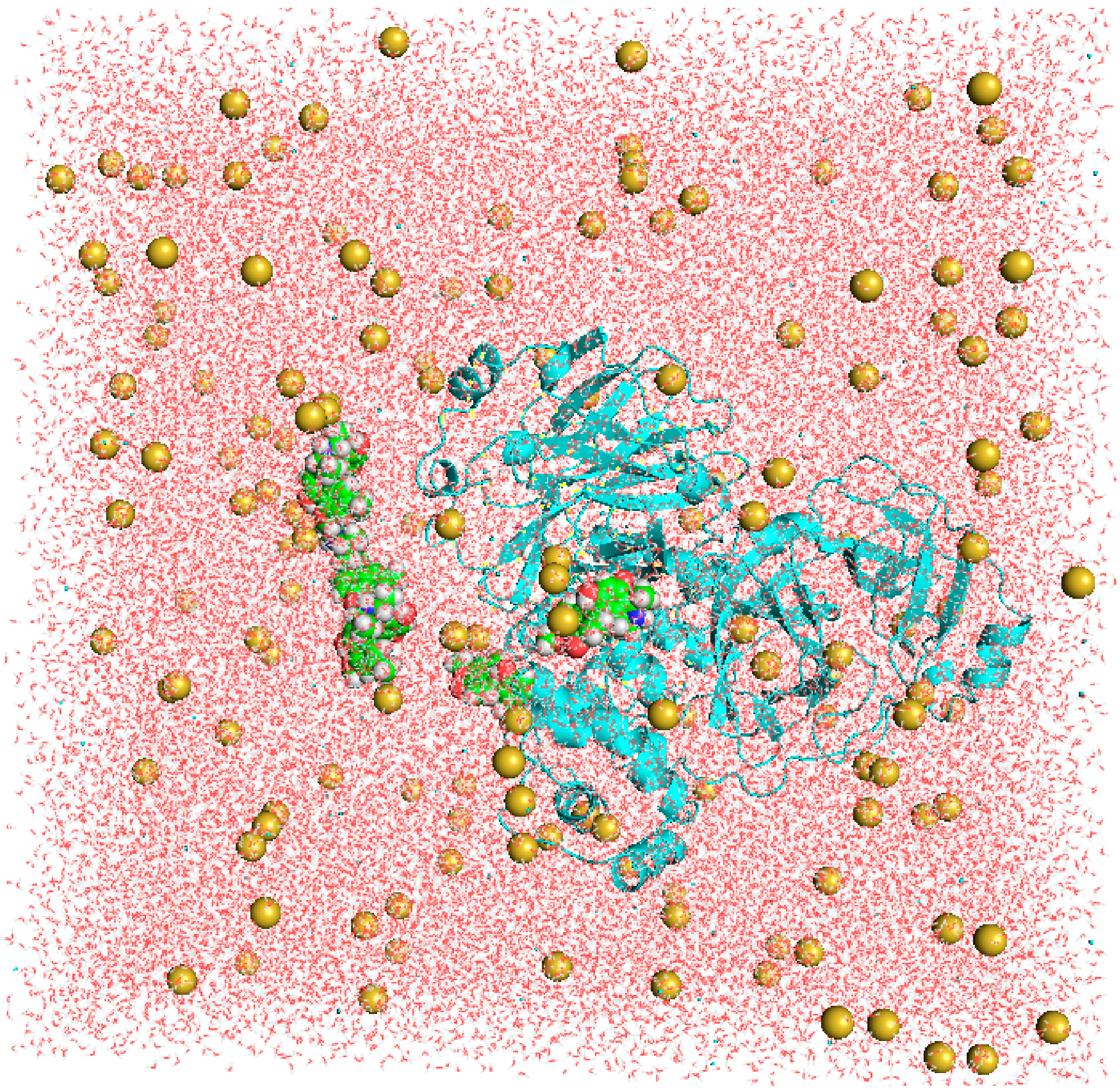

Figure S1. Initial model used in the GaMD simulations. Sinapic acid is shown as green spheres and M^{pro} as cyan ribbons. Golden-yellow balls represent the Na^{+} and Cl^{-} ions. A video showing the bindig event of sinapic acid inside the active site of M^{pro} can be found on the Zenodo website: https://zenodo.org/record/8175715.

Antiviral assay

Virus and cells

Vero-E6 cells were kept alive at 37 °C and 5% CO_2 in Dulbecco's Modified Eagle's medium (DMEM), which contains 2% penicillin/streptomycin and 10% bovine serum (BS) (Invitrogen). Cells were placed into tissue culture flasks 24 hours prior to infection with the hCoV-19/Egypt/NRC-3/2020 isolate in an infection medium containing 4% BS and 1% trypsin treated with L-1-tosylamido2-phenylethyl chloromethyl ketone (TPCK) in DMEM with 2% penicillin/streptomycin to produce the virus stock.

MTT cytotoxicity assay

The test compounds were diluted to working solutions with DMEM from stock solutions in 10 % DMSO with ddH_2O to determine the IC_{50} used for the compounds' initial antiviral screening. The cytotoxic effect of the test compounds was assessed in Vero-E6 cells using the previously described 3-(4,5-dimethylthiazol-2-yl)-2,5-diphenyltetrazolium bromide (MTT) technique [8] with minor modifications. The cells were incubated for 24 hours at 37 °C with 5% CO_2 after being placed in 96-well plates (100 µL/well at a density of 3105 cells/mL). After 24 hours, cells were exposed to different concentrations of the tested agents in triplicate. After another 24 hours, the supernatant was removed, and the cell monolayers were cleaned three times with sterile 1 PBS. Each well was given 20 µL of the 5 mg/mL stock MTT solution before being incubated for 4 hours at 37 °C. The formed formazan crystals were dissolved in 200 µL of acidified isopropanol (0.04 µM HCl in 100% isopropanol ¼ 0.073 mL HCL in 50 mL isopropanol). The absorbance of formazan solutions was then calculated using a microplate reader at a maximum wavelength of 540 nm. The percentage of cytotoxicity in comparison to untreated cells was determined using the formula below:

$$\%\text{Cytotoxicity} = \frac{(Absorbance\ of\ cells\ without\ treatment - absorbance\ of\ cells\ with\ treatment) x\ 100}{Absorbance\ of\ cells\ without\ treatment}$$

Viral replication assay

Vero cells were plated at a density of 4×10^4/well of a 24-well plate. Twenty-four hours later, the cells were infected in the SARS CoV-2 strain (the hCoV-19/Egypt/NRC-3/2020) at a multiplicity of infection (MOI) of 0.5. The inoculum was removed after 1 h and replaced by fresh medium complemented with different concentrations of the test compound. Virus RNA concentration in supernatant was measured by real-time PCR during the exponential growth phase of the viruses (i.e., after 2 days). For RNA preparation, a simple and inexpensive method (Boom et al., 1990) was adopted. A 140-µl aliquot of supernatant was mixed with 560 µl chaotropic lysis buffer (Qiagen) and incubated at room temperature for 15 min. The lysate was added to 100 mg diatomaceous silica (Sigma–Aldrich) suspended in 560 µl ethanol and incubated with agitation for 30 min at room temperature. The diatomaceous silica was pelleted by centrifugation and the pellet was washed with 500 µl AW1 buffer (Qiagen), subsequently with 500 µl AW2 buffer (Qiagen), and finally with 400 µl acetone. The pellet was dried at 56 ◦C and the RNA eluted with 100 µl water. Quantitative real-time PCR assays were performed with the purified RNA based on previously published protocols (Drosten et al., 2003; Asper et al., 2004). The PCR reaction conditions were as the following: 25-µl reaction based on Superscript II RT/Platinum Taq polymerase one-step RT-PCR kit (Invitrogen): 5µl RNA, 1× buffer, 3.6 mM additional $MgSO_4$, 0.6 µl enzyme mixture, 240 nM probe BNITMSARP (FAM-TCG TGC GTG GAT TGG CTT TGA TGT-TAMRA), 200 nM primer BNITMSARS1 (TTA TCA CCC GCG AAG AAG CT), and 200 nM primer BNITMSARAs2 (CTC TAG TTG CAT GAC AGC CCT C). The PCR cycling condition: 7000 SDS machine (Applied Biosystems): 15 min at 45 ◦C; 3 min at 95 ◦C; 40 cycles with 15 s at 95 ◦C and 30 s at 58 ◦C with fluorescence measured at 58 ◦C. The concentrations required to inhibit virus replication by 50% (IC_{50}) were calculated by fitting a sigmoidal curve to the data following logarithmic transformation of the drug concentration.

References

[1] E. Chow, C.A. Rendleman, K.J. Bowers, R.O. Dror, D.H. Hughes, J. Gullingsrud, D.E. Shaw, Desmond performance on a cluster of multicore processors, DE Shaw Research Technical Report DESRES/TR (2008) 2008-01.

[2] M. Bergdorf, S. Baxter, C.A. Rendleman, D.E. Shaw, Desmond/GPU Performance as of October 2015, DE Shaw research (2015).

[3] L. Wang, Y. Wu, Y. Deng, B. Kim, L. Pierce, G. Krilov, R. Abel, Accurate and reliable prediction of relative ligand binding potency in prospective drug discovery by way of a modern free-energy calculation protocol and force field, J. Am. Chem. Soc. 137 (2015) 2695-2703.

[4] J.C. Phillips, R. Braun, W. Wang, J. Gumbart, E. Tajkhorshid, E. Villa, C. Chipot, R.D. Skeel, L. Kale, K. Schulten, Scalable molecular dynamics with NAMD, J. Comput. Chem. 26 (2014) 1781–1802.

[5] S. Kim, H. Oshima, H. Zhang, N.R. Kern, S. Re, J. Lee, B. Roux, Y. Sugita, W. Jiang, W. Im, CHARMM-GUI free energy calculator for absolute and relative ligand solvation and binding free energy simulations, J. Chem. Theory Comput. 16 (2020) 7207–7218.

[6] S.T. Ngo, N.M. Tam, M.Q. Pham, T.H. Nguyen, Benchmark of popular free energy approaches revealing the inhibitors binding to SARS-CoV-2 Mpro, J. Chem. Inf. Model 61 (2021) 2302–2312.

[7] Y.T. Pang, Y. Miao, Y. Wang, J.A. McCammon, Gaussian accelerated molecular dynamics in NAMD, J. Chem. Theory Comput. 13 (2017) 9–19.

[8] Mosmann, T. (1983). Rapid colorimetric assay for cellular growth and survival: Application to proliferation and cytotoxicity assays. Journal of Immunological Methods, 65(1–2), 55–63. https://doi.org/10.1016/0022- 1759(83)90303-4.

[9] Boom, R. C. J. A., Sol, C. J., Salimans, M. M., Jansen, C. L., Wertheim-van Dillen, P. M., & Van der Noordaa, J. P. M. E. (1990). Rapid and simple method for purification of nucleic acids. *Journal of clinical microbiology*, *28*(3), 495-503.

[10] Drosten, C., Günther, S., Preiser, W., Van Der Werf, S., Brodt, H. R., Becker, S., ... & Doerr, H. W. (2003). Identification of a novel coronavirus in patients with severe acute respiratory syndrome. *New England journal of medicine*, *348*(20), 1967-1976.

[11] Asper, M., Sternsdorf, T., Hass, M., Drosten, C., Rhode, A., Schmitz, H., & Günther, S. (2004). Inhibition of different Lassa virus strains by alpha and gamma interferons and comparison with a less pathogenic arenavirus. *Journal of virology*, *78*(6), 3162-3169.
